# Genetic alterations in benign prostatic hyperplasia patients

**DOI:** 10.3205/000257

**Published:** 2017-11-27

**Authors:** Hanaa Mahmoud Mohamed, Magdy Sayed Aly, Tarek Dardeer Hussein

**Affiliations:** 1 Cell Biology and Genetics Division, Zoology Department, Faculty of Science, Beni-Suef University, Beni-Suef, Egypt; 2 Department of Zoology, Faculty of Science, Cairo University, Cairo, Egypt

**Keywords:** benign prostatic hyperplasia, BPH, fluorescence in situ hybridization, FISH, genes, amplification, deletion

## Abstract

**Background:** Benign prostate hyperplasia (BPH) is a classical age-related disease of the prostate, present in 20% of men at the age of 40 years with progression to 70% by the age of 60 years. BPH is associated with various lower urinary tract symptoms, which affect their day-to-day life.

**Materials and methods:** Our objective was to evaluate the association between HER-2/neu, c-myc, p53, and clinicopathological variables in 45 patients diagnosed with benign prostatic hyperplasia using fluorescence in situ hybridization (FISH). The patients underwent transurethral prostate resection to address their primary urological problem. All patients were evaluated by use of a comprehensive medical history and rectal digital examination. The preoperative evaluation also included serum prostate specific antigen (PSA) measurement and ultrasonographic measurement of prostate volume.

**Results:** The mean (± standard deviation) age of the 45 patients was 69.65 ± 8.97 years. The mean PSA value of the patients was 9.25 ± 5.12 ng/mL. The mean prostate volume was 65.46 ± 11.43 mL. Amplification of HER-2/neu was seen in 4/45 (8.9%) cases and amplification of c-myc was seen in 5 of 45 (11.1%) cases; both genes were not associated with adverse clinicopathological variables. Deletion of p53 was seen in 20/45 (44.4%) cases. p53 gene was significantly associated with a severe AUASI (American Urological Association Symptom Index) score.

**Conclusion:** In this study, we discussed important genetic markers in benign prostatic hyperplasia patients which may, in the future, be used as markers for diagnosis and prognosis, as well as targets for therapeutic intervention.

## Zusammenfassung

**Hintergrund:** Benigne Prostatahyperplasie (BPH) ist eine klassische altersbedingte Erkrankung der Prostata, die in 20% der Männer im Alter von 40 Jahren auftritt mit einer Progression zu 70% im Alter von 60 Jahren. BPH ist mit verschiedenen Symptomen der unteren Harnwege assoziiert, die Auswirkungen auf das Alltagsleben haben.

**Materialien und Methoden:** Unser Ziel war es, die Verbindung zwischen HER-2/neu, c-myc, p53 und klinisch-pathologischen Variablen bei 45 Patienten mit benigner Prostatahyperplasie unter Anwendung von Fluoreszenz-in-situ-Hybridisierung (FISH) zu bewerten. Bei den Patienten wurde eine transurethrale Prostataresektion durchgeführt, um ihr primäres urologisches Problem zu behandeln. Alle Patienten wurden anhand einer umfassenden Anamnese und einer rektalen digitalen Untersuchung beurteilt. Die präoperative Auswertung umfasste auch die Messung des prostataspezifischen Antigens (PSA) sowie die sonographische Messung des Prostatavolumens.

**Ergebnisse:** Das mittlere (± Standardabweichung) Alter der 45 Patienten betrug 69,65 ± 8,97 Jahre. Der mittlere PSA-Wert der Patienten betrug 9,25 ± 5,12 ng/ml. Das mittlere Prostatavolumen betrug 65,46 ± 11,43 ml. Amplifikation von HER-2/neu wurde in 4/45 (8,9%) Fällen und Amplifikation von c-myc in 5 von 45 (11,1%) Fällen gesehen; beide Gene waren nicht mit nachteiligen klinisch-pathologischen Variablen assoziiert. Eine Deletion von p53 wurde in 20/45 (44,4%) Fällen beobachtet. Das p53-Gen war signifikant mit einem hohen AUASI (American Urological Association Symptom Index)-Score assoziiert.

**Fazit:** In dieser Studie wurden wichtige genetische Marker bei benigner Prostatahyperplasie diskutiert, die möglicherweise in Zukunft als Marker für Diagnose und Prognose sowie als Ansatzpunkte für therapeutische Intervention dienen können.

## Introduction

Benign prostatic hyperplasia (BPH) is one of the most common diseases found in adult men [1]. BPH is characterized by the proliferation of smooth muscle cells and epithelial cells within the prostatic transition zone [1]. The exact etiology and mechanisms underlying BPH development and progression are not fully understood [1], [2]. Benign prostatic hyperplasia mostly develops in a small region, the transition zone, close to the urethra [3]. Prostatic cancer and benign prostatic hyperplasia are often found at the same time in elderly men; however, the relation between the two has been controversial since their earliest descriptions [4]. The American Urological Association Symptom Index (AUASI) score is a self-administered questionnaire, used to assess the severity of three storage symptoms (frequency, nocturia, urgency) and four voiding symptoms (feeling of incomplete emptying, intermittency, straining, and a weak stream) and to help diagnose BPH. How frequently the patient experiences each symptom is rated on a scale of 1 to 5 [5].

Cytogenetic information on malignant and benign prostatic tumors is limited because of the difficulties in culturing prostatic epithelial cells. Although improvements in existing techniques have been achieved [6], 75% of cytogenetically investigated prostate tumors, almost exclusively adenocarcinomas, have shown a normal male karyotype, and no consistent chromosome change has been associated with this malignancy [7]. 

Few studies have been conducted on chromosomal abnormalities and gene polymorphisms in patients with BPH [8], [9], [10], [11], therefore information regarding cytogenetic changes in these patients is scarce. 

In the current study, we assessed the genetic alterations of HER-2/neu, c-myc and p53 genes using fluorescence in situ hybridization (FISH) in Egyptian benign prostatic hyperplasia patients and investigated the prognostic role of the three markers and their relation to each other and to demonstrate their relation to the classical clinicopathological factors.

## Materials and methods

### Patients 

The study protocol was reviewed and approved by the Ethical Committee of National Cancer Institute, Cairo University (IRB No. 00004025) and (FWA No. 00007284). Forty-five consecutive samples from patients diagnosed with BPH (n= 45) were obtained from the archived collection at the National Cancer Institute, Cairo, Egypt. The age of the patients ranged from 42 to 89 years. The patients were preoperatively considered to have BPH and underwent surgery for their primary urological problem. Preoperative evaluation consisted of rectal examination, transrectal sonography, prostate specific antigen (PSA) determination, and prostate biopsies, if warranted. Benign hyperplastic prostate tissue samples were obtained from adenomectomy (A) and transurethral resection of prostate (TURP) or from cystoprostatectomy (CP) for invasive bladder cancer. 

### FISH analysis

For each specimen, 5 µm tissue sections were cut and placed on positively charged microscope slides. The blocks were characterised by staining one out of 10 serial sections through the block with haematoxylin and eosin (H&E) followed by examination by an expert pathologist to confirm that no histological features of adenocarcinoma or prostatic intraepithelial neoplasia (PIN) are present. The specimen slides used for the FISH assay procedure were within 10 serial sections of the respective H&E-stained slide to assure minimal separation of the areas examined by FISH from the areas evaluated by histopathology. Three slides were set for each case, and each slide was used for hybridization with a probe cocktail, one probe specific for the gene under investigation and the other specific for the chromosome containing the gene. For example, the HER-2/neu probe cocktail consists of a HER-2/neu probe (Spectrum Orange) and a chromosome 17 centromere-specific probe (Spectrum Green). All probe cocktails for HER-2/neu, c-myc, and p53 genes were purchased from Abbott Molecular (Des Plaines, IL). Slides were treated in xylene twice for 10 minutes to remove paraffin, denatured in 70% formamide at 70°C for 3 minutes, and dehydrated in a cooled alcohol series of 70, 80, 90, and 100% for 2 minutes each. Ten microliters of the denatured probe were placed on each slide, covered with a glass coverslip, and sealed with rubber cement. Hybridization was continued overnight at 37°C. Slides were washed twice in 50% formamide at 47°C for 2 minutes and twice in 2x standard saline citrate at room temperature for 2 minutes. The slides were stained with DAPI as a counter stain and scanned using a 90i Nikon fluorescent microscope (Chroma Technology, Brattleboro, VT) at a magnification of 1000X. Only intact, non-overlapping nuclei were evaluated; positive signals were required to be bright and of approximately equal intensity among the nuclei. Genes were considered amplified if they showed a gene/ centromere ratio of more than 2.2 after counting at least 100 nuclei. Ratio under 2.0 was considered unamplified [12],[13]. For p53, we also defined the FISH score as the percentage of cells for which the nuclei had lost at least one signal. The specificity of the probes and the validity of this method were checked by dual-color FISH using normal prostate sections from five individuals and normal human male peripheral lymphocytes.

### Statistical analysis

Data were analyzed using SPSS version 23. Data were expressed as mean ± SD. Bivariate correlations were tested using Pearson’s correlation coefficient for parametric data or Spearman’s rho for non-parametric data. Proportions were compared using chi-squared test. Two-tailed *p*-values were considered statistically significant if they were less than 0.05.

## Results

To determine the efficiency of in situ hybridization, normal prostate sections from five individuals and peripheral lymphocytes from normal human male were hybridized with the three probe cocktails. In most of the cells, two orange signals for the single-copy probe (MYC, ERBB2, and TP53), and two green signals for chromosome 17 or 8 were observed. 

Table 1 (Tab. 1) shows the major characteristics of the patients under the study. A total of 45 benign prostate tumor patients were included. The mean age was 69.55 ± 8.97 with a range of 42–89 years. PSA level was 9.25 ± 5.12 ng/mL with a range of 3.28–29.60 ng/mL. The volume of prostate was 65.46 ± 11.43 mL with a range of 47.0–88.0 mL. The PSAD, prostate specific antigen density, was 0.14 ± 0.09 ng/ml/cm^3^ with a range of 0.04–0.54 ng/ml/cm^3^. With respect to surgery techniques, 26 patients (57.8%) had adenotectomy, 11 (24.4%) had transurethral resection of prostate, 7 (15.6%) had cystoprostatectomy and one patient (2.2%) had radical retropubic prostatectomy. With respect to the previous treatments, 33 out of 45 (73.3%) had no treatment while 11(24.4%) had treatment related to transitional cell carcinoma of the bladder. With respect to the severity of BPH, the American Urological Association Symptom Index (AUASI) was used. Three cases out of 45 (6.7%) were mild, 14 (31.1%) were moderate and 28 (62.2%) were severe.

Using FISH analysis, we found that 38 of 45 cases (84.4%) patients were disomic, 5 (11.1%) were monosomic, and 2 cases (4.4%) were polysomic for chromosomes 17 in at least 80% of their cells. Thirty-nine out of 45 patients (86.7%) were disomic, 4 (8.9%) were monosomic, and two (4.4) were polysomic for chromosome 8 in at least 80% of their cells (Table 2 (Tab. 2)). 

**Figure 1:**
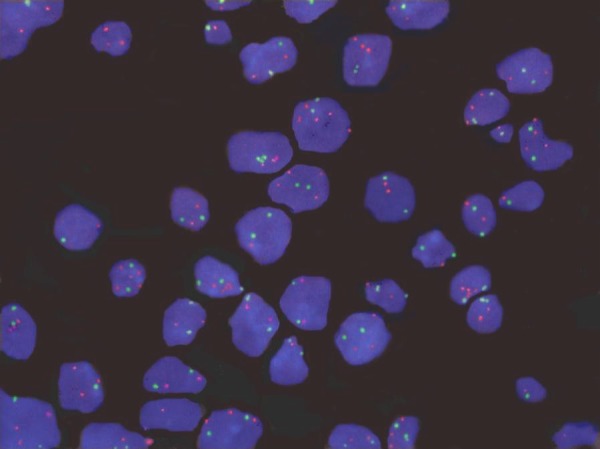
Hybridization of BPH sections with probes specific to Her-2/neu gene (orange) and chromosome 17 centromere (green). Two signals for chromosome 17 and more than two signals for the Her-2/neu gene can be seen.

**Figure 2:**
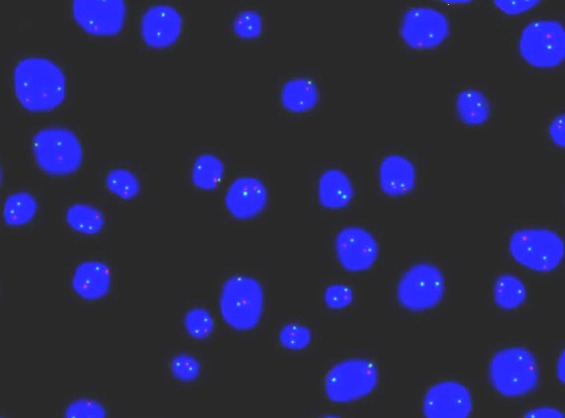
Representative dual-color fluorescence in situ hybridization experiments of BPH patient with centromeric probe of chromosome 17 (green) and p53 gene (orange) showing two signals of chr17 and two or one or zero p53 signal (s)

Out of the 45 patients, 4 (8.9%) had amplification of HER-2/neu gene (Figure 1 (Fig. 1)), 3 (6.7%) were lacking one HER-2/neu gene and (84.4%) had the normal two copies of the gene present in at least in 80% of the cells. Also, out of the 45 patients, 5 (11.1%) patients had amplification of c-myc gene, 3 (6.7%) had one c-myc gene deleted and 37 (82.2%) had the normal two copies of the gene present in at least 80% of the cells. For the p53 gene, 20 patients (44.4%) had evident deletion of at least one copy of the gene in a high proportion of cells (Figure 2 (Fig. 2)), whereas 25 (55.6%) patients had the normal two copies of the gene in at least 80% of the cells (Table 3 (Tab. 3)).

### Previous treatment of patients

Table 4 (Tab. 4) shows a comparison between patients previously treated from TCC and those without treatment. For chromosome 17, loss or gain was detected in 6 out of 11 (45.5%) patients treated, whereas, in patients with no treatment, only 2 patients out of 33 (6%) showed abnormal number of copies of chromosome 17; the difference in the proportion was significant (p=0.002). For chromosome 8, the proportion of abnormal cases in patients treated was 18.2% which was more than non-treated patients (12.1%) but insignificantly. For gene signals, HER-2/neu showed significant increase in the proportion of abnormalities in patients treated (45.5%) than those not treated (12.1%, p=0.01). For p53 and c-myc copy numbers, no significant difference could be detected.

### AUASI score

Table 5 (Tab. 5) illustrates the association between the AUASI score and the copy number of chromosomes and gene signals. p53 gene copy number was the only factor significantly associated with the AUASI score where 71.4% of p53 abnormal copy number was focused in the severe cases whereas the mild and moderate cases had the normal number of gene copies. 

### Correlation between the alteration of genes and clinicopathological characteristics.

**Figure 3:**
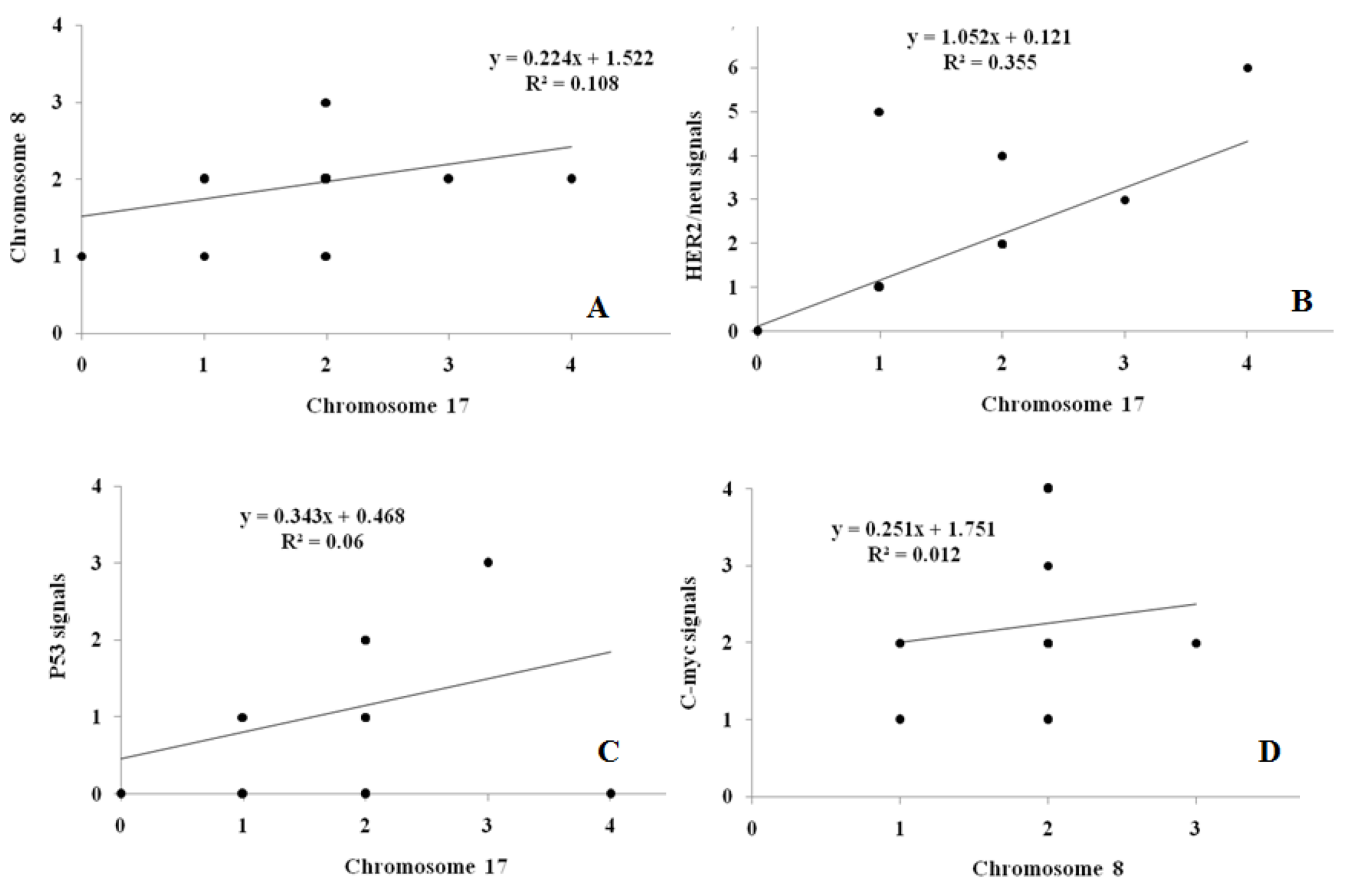
Correlation between chromosome copy number and gene signals in the studied patients. A: correlation between ch 17 and ch 8 (r=0.315, p=0.035). B: Correlation between ch 17 and HER2/neu signals (r=0.640, p=0.001). C: Correlation between ch 17 and P53 signals (r=0.304, p=0.042). D: Correlation between ch 8 and C-myc signals (r=0.153, p=0.315, NS).

Univariate analysis revealed no association between aneuploidy and clinicopathological characteristics of patients. Univariate analysis failed to reveal any significant association between the oncogene copy number and clinicopathologic variables examined. Table 4 (Tab. 4) shows the correlation between the different variables. Significant correlations were observed between chromosome 8 and chromosome 17 copy numbers (r=0.315, p<0.05, Figure 3A (Fig. 3)), between chromosome 17 and p53 signals (r=0.304, p<0.05, Figure 3B (Fig. 3)) and between chromosome 17 and HER-2/neu signals (r=0.649, p<0.01, Figure 3C (Fig. 3)). No statistically significant correlation could be detected chromosome 8 copies and HER-2/neu signals (r=0.289, p>0.05, Figure 3D (Fig. 3)). With respect to the severity of BPH cases (AUASI score), significant positive correlations were observed with the age (r=0.424, p<0.01), PSA (r=0.673, p<0.01), PV (r=0.322, p<0.05) and PSAD (r=0.534, p<0.01). Negative correlation was detected between the severity of cases and P53 copy numbers (r=–0.591, p<0.01, Table 6 (Tab. 6)).

## Discussion

Chromosomal changes in normal prostatic tissue and in BPH tissue have been investigated in few studies in contrast to prostate cancer tissues. Miyauchi et al. [14] studied 10 cases of BPH were karyotyped by the G-banding method. Structural analysis disclosed 2 cases of BPH were diploid whereas normal male karyotypes were seen in 6 BPH. Trisomy of chromosomes 7 and 16 were observed in 2 BPH in the same study. Aly et al. [9] found that out of 28 samples of BPH, loss of the Y chromosome was the most common chromosome change, followed by trisomy 7. Visakorpi et al. [15] demonstrated that out of 10 BPH patients, BPH specimens were diploid by DNA flow cytometry and showed no numerical chromosomal aberrations by FISH technique. 

Balachandar et al. [16], in 2008, studied 63 BPH and 18 prostate cancer patients. Deletions, translocations, inversions and mosaics were the major chromosomal aberrations observed in the patients regardless their age. Chromosome 1, 7, 16 and Y were affected in BPH patients. In 2010, Balachandar et al. [8] reported major chromosomal aberrations like deletion, translocation, inversion in chromosomes 1, 6, 8, 13, 16, 18 in blood samples of BPH patients. Recently, Altok et al. [17] demonstrated that chromosomal abnormalities were noted in 5 of the 53 cases (9.4%). Loss of the Y chromosome was the most frequent chromosomal abnormality and was observed in three patients (5.7%). 

In our study, we successfully investigated dual-color FISH cytogenetic analysis on 45 patients with histologically proven BPH using two oncogenes and one tumor suppressor gene. We wished to identify genetic alterations in BPH and to estimate their eventual correlations with the genetic alterations of prostatic adenocarcinoma. If the genetic alterations of the adenocarcinoma (epithelial in origin) are also present in nonmalignant BPH (which usually do not evolve to malignancy) and in nonepithelial prostatic tissues, these changes cannot be considered relevant to the origin of prostatic adenocarcinoma.

HER-2/neu gene, a member of the epidermal growth factor receptor (EGFR) family, encodes a transmembrane phosphoprotein. The prognostic significance of HER-2/neu in breast cancer is well established [18]. Controversy remains regarding the significance HER-2/neu in prostate cancer. Edwards et al. [19] showed HER-2/neu amplification in 8% of prostate cancers (using FISH and IHC methods used for HER-2/neu diagnosis in breast cancer) suggesting that HER-2/neu is not involved in early prostate cancer and agrees with the data of Mark and colleagues who assessed HER-2/neu amplification using identical methods [20]. Ross and colleagues reported 41% of prostate tumors with an amplified HER-2/neu [21]. Three other studies found no HER-2/neu amplification [22], [23], [24], although this may be due to sampling errors. 

In our study, although the statistical analysis failed to reveal any association between HER-2/neu gene amplification and clinicopathological data, FISH was demonstrated to be an appropriate technique for this type of study. Using FISH, amplification of the HER-2/neu gene was found in 8.9% of our samples. This rate is close to that detected in patients with cervical carcinoma and melanoma (5%–20%). This is in contrast to Edwards et al. [19] who found no abnormalities of HER-2/neu gene copy number, chromosome 17 copy or HER2 gene : chromosome ratio in their study of 28 BPH tissues.

The c-myc oncogene is a transcription factor that has pleiotropic effects on cell growth and differentiation. Amplification or overexpression of c-myc was detected in many human cancers including prostate cancer. Specifically, c-myc mRNA levels were found to be significantly higher in malignant tissues compared to BPH [25], [26]. Amplification of the c-myc locus has been found in some primary prostate tumors and in lymph node metastases [27], [28]. Other studies reported no significant amplification of c-myc expression [29], [30]. Fox et al. [31] showed by immunohistochemical that, detection of c-myc has not provided useful prognostic information for patients with early-stage prostate cancer.

In our study, the c-myc oncogene was amplified in 11.1% of BPH samples. Bivariate analysis failed to reveal any significant association between oncogene amplification and the clinicopathologic variables examined. Thus, further study of c-myc gene is required to clarify the role of this oncogene in prostate cancer and in BPH.

p53 gene is the most frequently mutated gene in human cancers. The main function of p53 gene is the prohibition of entrance into the synthetic phase of the cell cycle and the promotion of apoptosis in cells that are incompetent or have damaged DNA [32], [33]. In primary prostate cancer, a relatively low incidence (10–20%) of p53 gene mutations has been described, however, in advanced stages of the disease, the p53 is mutated in 42% of the cases and it is associated with bone metastases and androgen-independent disease [34], [35]. Abnormal p53 expression correlates with high histological grade, high stage and clinical progression of the disease [36] while, it is also correlated with reduced survival after radical prostatectomy [37] and disease onset modulation [38]. p53 mutations are detectable in approximately 19.0% in patients with benign hyperplasia [39].

In our study, deletion of the p53 gene was the most significant finding. Twenty patients (44.4%) had evidence of deletion of at least one copy of p53 in a high proportion of the cells. Significant correlations were detected between chromosome 17 and p53 signals (r=0.304, p<0.05). p53 gene copy number was significantly associated with the AUASI score where 71.4% of p53 abnormal copy number was detected in the severe cases whereas the mild and moderate cases had the normal copy number. 

O'Leary et al. [40] studied 4325 men with lower urinary tract symptoms caused by BPH had moderate to severe symptoms (AUASI score >12). Roehrborn et al. [41] found that men with lower urinary tract symptoms (LUTS) & clinical BPH and no history of urinary retention, the AUASI score are useful parameters for clinicians in identifying patients at risk for future prostate surgery. 

## Conclusion

This is the first report to demonstrate genetic alterations in Egyptian patients with BPH. The most important alteration detected is for p53; it showed abnormal copy number in the majority of our patients. Consequently, deletion of p53, when evident in prostatic adenocarcinoma, cannot be considered specific of or relevant for the genesis of this tumor, but although the patients studied had no evidence of carcinoma, they may still develop prostate cancer or may have a latent disease that was not detected. Indeed, future studies including larger databases of patients, which integrate clinical, pathologic, and molecular oncogene parameters are strongly recommended to identify the possible genetic abnormalities underlying BPH etiopathogenesis. The merging of these data may provide the clinician with an enhancement of prognostic information that accurately predicts the aggressive phenotype for benign prostatic hyperplasia. 

### Clinical practice points

- No previous reports have been obtained for genetic alterations studies performed on Egyptian BPH patients.
- We recorded 44.44% of deletion of p53 gene.
- The patients studied had no evidence of carcinoma, they may develop prostate cancer. Future studies are recommended to identify the possible genetic abnormalities underlying BPH etiopathogenesis.

## Notes

### Competing interests

The authors declare that they have no competing interests.

## Figures and Tables

**Table 1:**
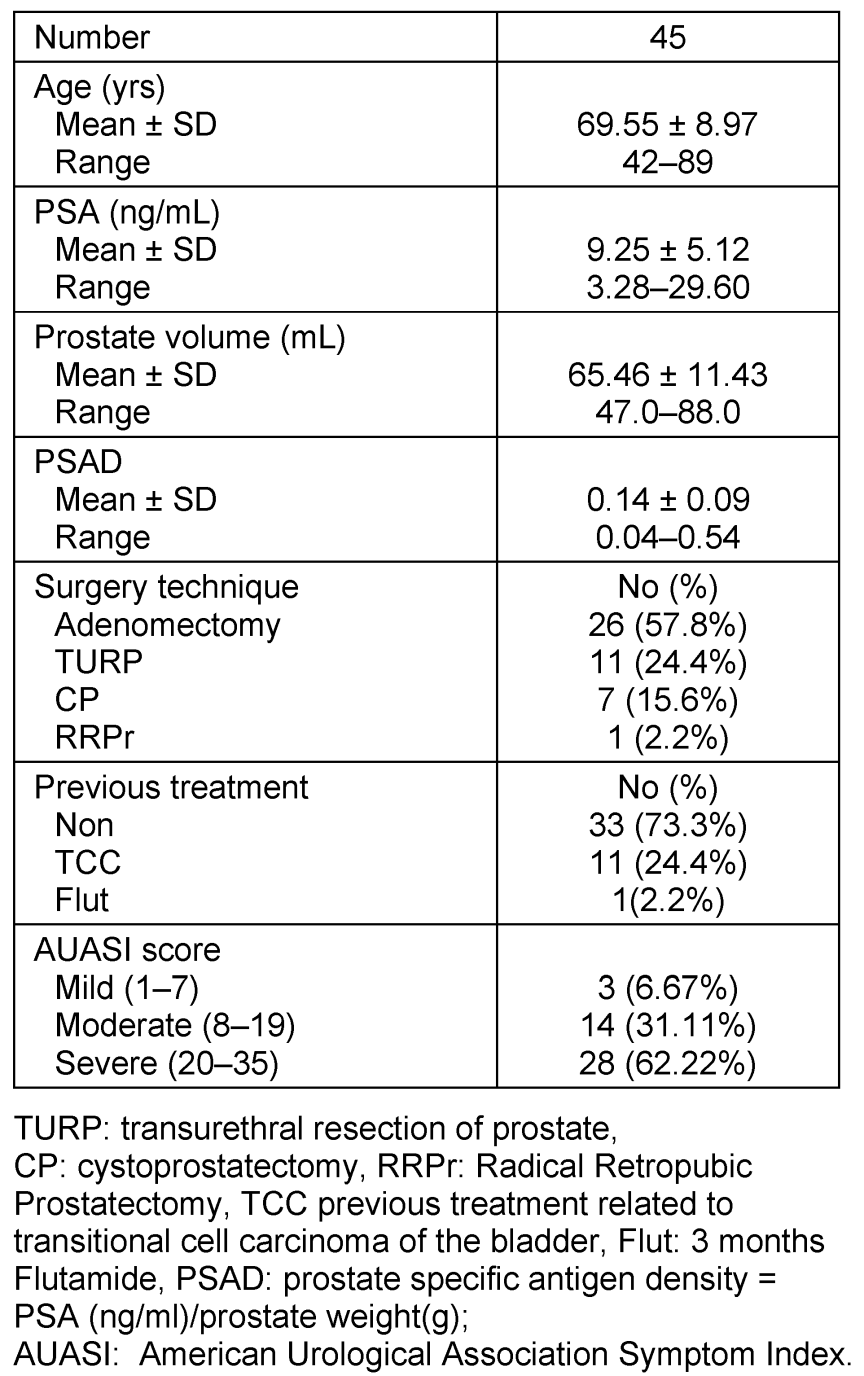
Major characters of benign prostatic hyperplasia patients

**Table 2:**
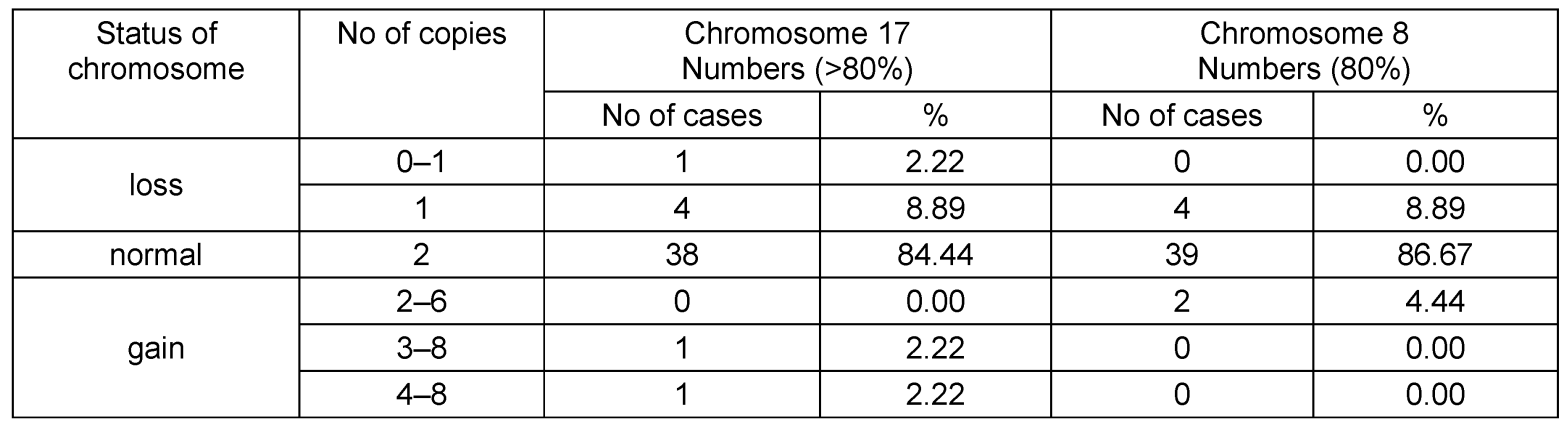
Chromosome 17 and 8 centromeres copy number

**Table 3:**
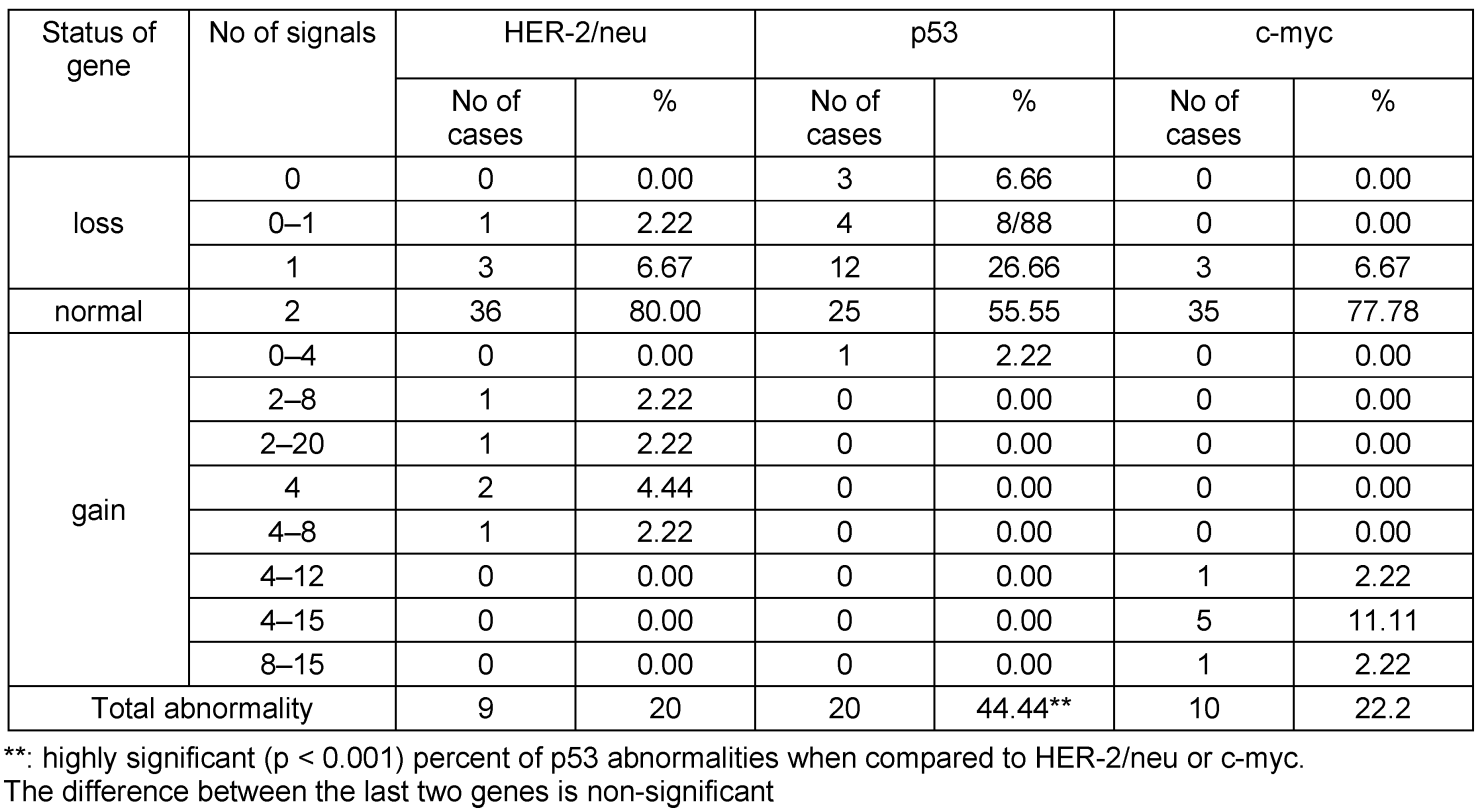
Number of HER-2/neu, c-myc, and p53 signals numbers per cell

**Table 4:**
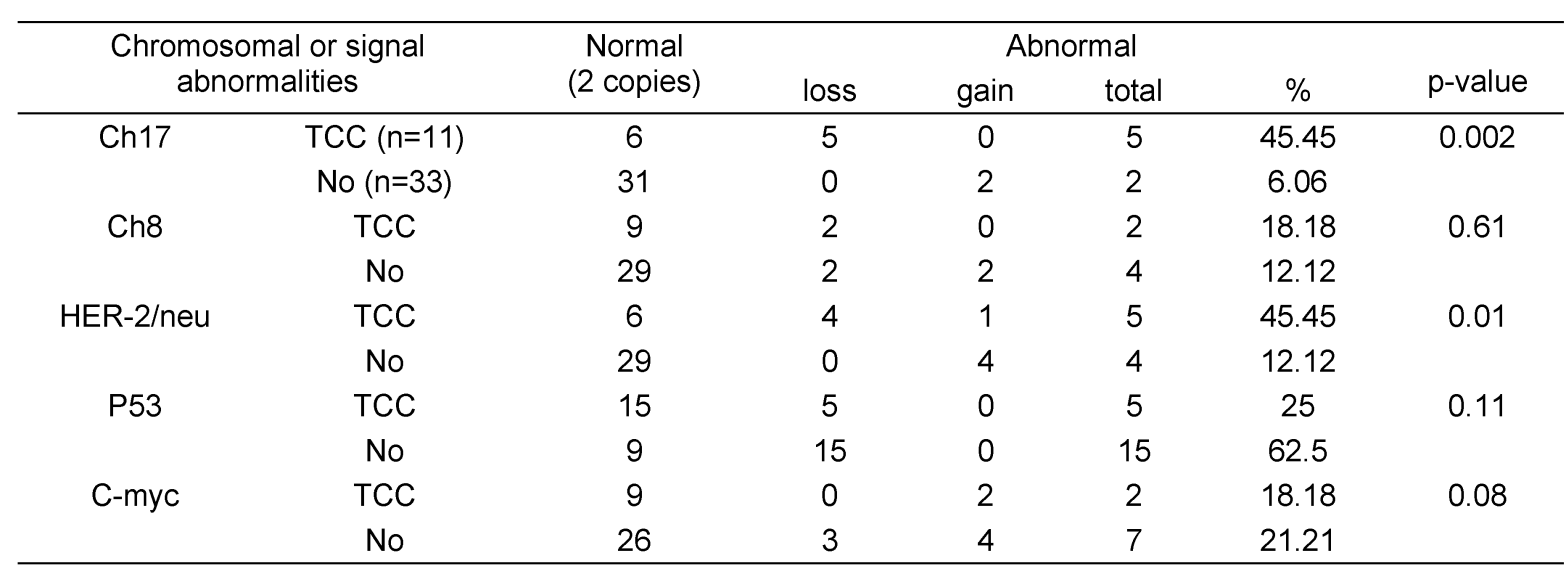
Association between the previous treatment and chromosomal and gene signal abnormalities

**Table 5:**
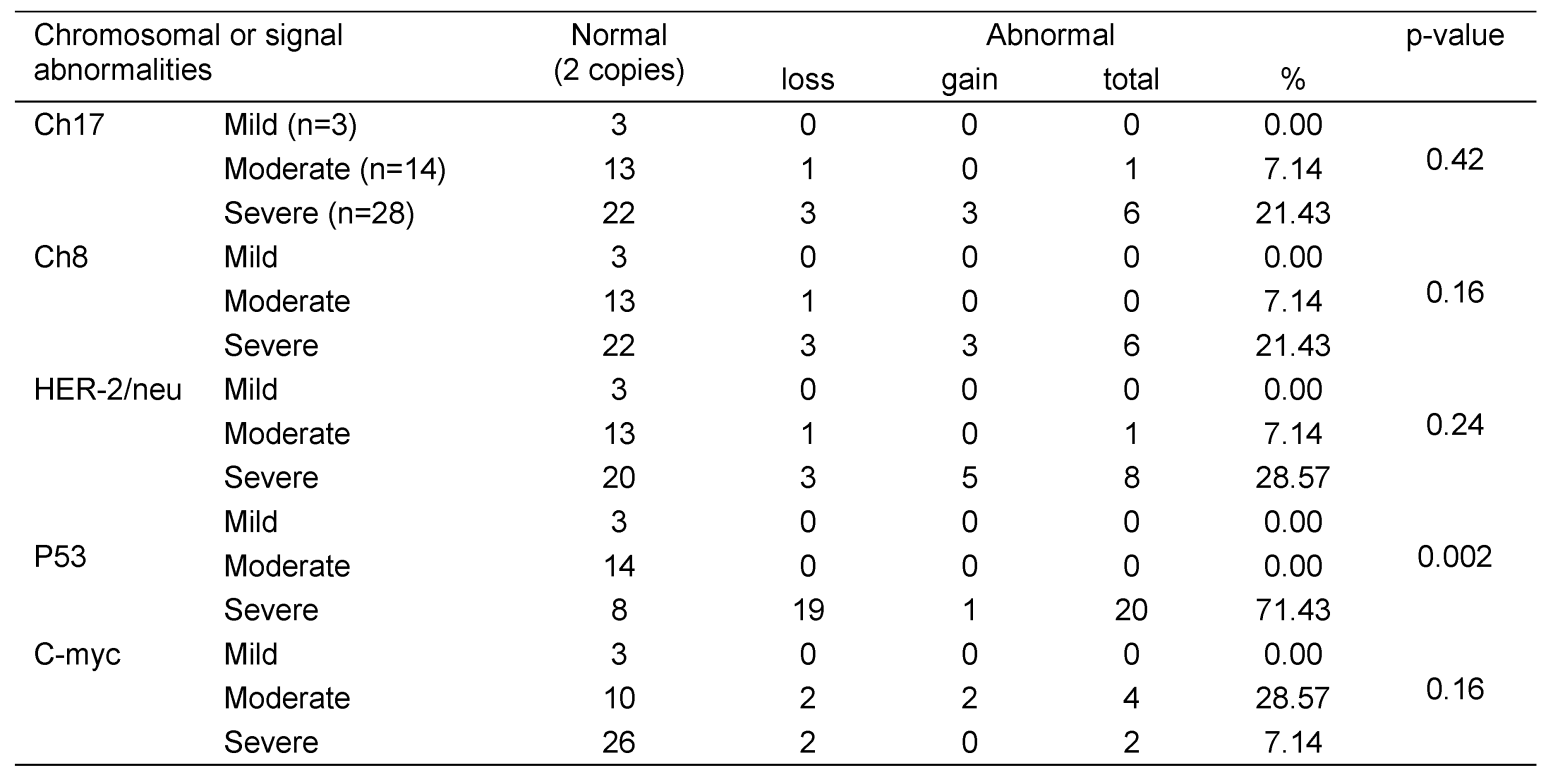
Association between the AUASI score and chromosomal and gene signal abnormalities

**Table 6:**
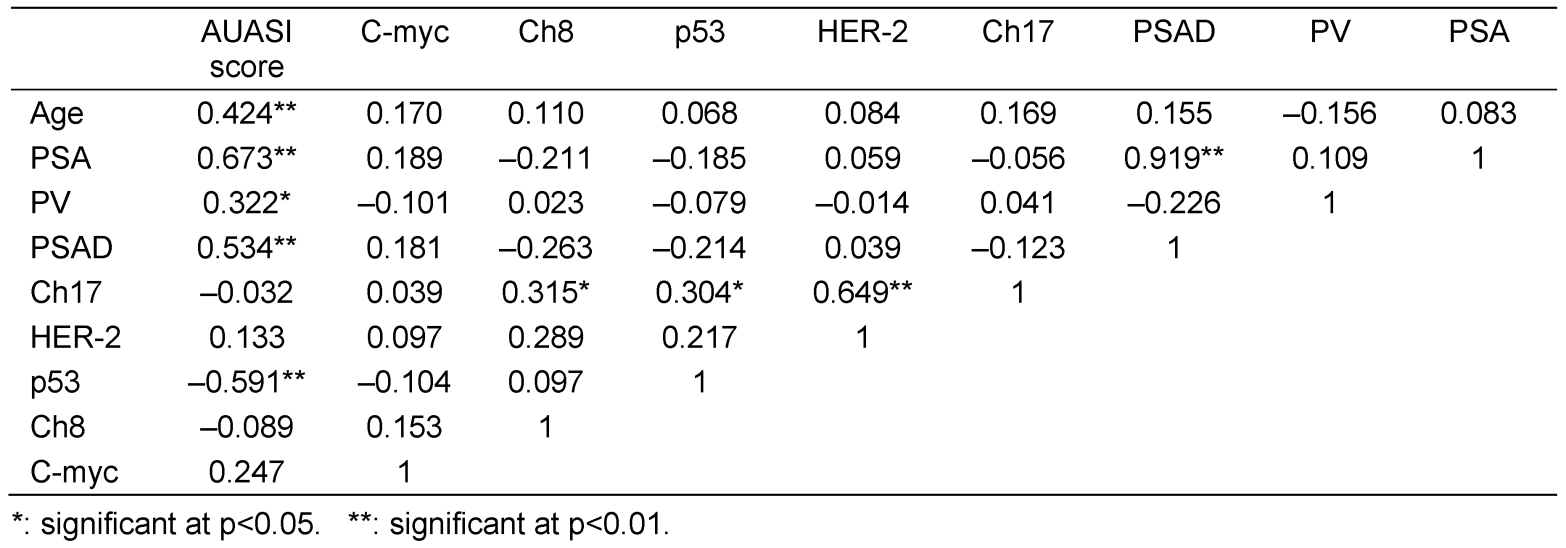
Correlation between age, PSA, prostate volume PSAD and chromosomal and signal abnormalities
